# Role of adjuvant chemotherapy in locally advanced rectal cancer with ypT0-3N0 after preoperative chemoradiation therapy and surgery

**DOI:** 10.1186/s12885-017-3624-7

**Published:** 2017-09-02

**Authors:** Chang Gon Kim, Joong Bae Ahn, Sang Joon Shin, Seung Hoon Beom, Su Jin Heo, Hyung Soon Park, Jee Hung Kim, Eun Ah Choe, Woong Sub Koom, Hyuk Hur, Byung Soh Min, Nam Kyu Kim, Hoguen Kim, Chan Kim, Inkyung Jung, Minkyu Jung

**Affiliations:** 1 Division of Medical Oncology, Department of Internal Medicine, Yonsei Cancer Center, 50-1 Yonsei-ro, Seodaemun-gu, Seoul, 120-752 South Korea; 2 Graduate School of Medical Science and Engineering, KAIST, Daejeon, South Korea; 3 Department of Radiation Oncology, Yonsei Cancer Center, Seoul, South Korea; 4 Department of Surgery, Yonsei Cancer Center, Seoul, South Korea; 5 Department of Pathology, Yonsei Cancer Center, Seoul, South Korea; 6 Division of Medical Oncology, Department of Internal Medicine, CHA Bundang Medical Center, Seongnam, South Korea; 7 Department of Biostatistics and Medical Informatics, Yonsei University College of Medicine, 50-1 Yonsei-ro, Seodaemun-gu, Seoul, 120-752 South Korea

**Keywords:** Rectal cancer, Adjuvant chemotherapy, Disease-free survival, Overall survival

## Abstract

**Background:**

We aimed to explore the clinical benefit of adjuvant chemotherapy (AC) with fluoropyrimidine in patients with ypT0-3N0 rectal cancer after preoperative chemoradiation therapy (CRT) followed by total mesorectal excision (TME).

**Methods:**

Patients with ypT0-3N0 rectal cancer after preoperative CRT and TME were included using prospectively collected tumor registry cohort between January 2001 and December 2013. Patients were categorized into two groups according to the receipt of AC. Disease-free survival (DFS) and overall survival (OS) were compared between the adjuvant and observation groups. To control for potential confounding factors, we also calculated propensity scores and performed propensity score-matched analysis for DFS and OS.

**Results:**

Of the 339 evaluated patients, 87 patients (25.7%) did not receive AC. There were no differences in DFS (hazard ratio [HR], 0.921; 95% confidence interval [CI], 0.562–1.507; *P* = 0.742) and OS (HR, 0.835; 95% CI, 0.423–1.648; *P* = 0.603) between the adjuvant and observation groups. After propensity score matching, DFS (HR, 1.129; 95% CI, 0.626–2.035; *P* = 0.688) and OS (HR, 1.200; 95% CI, 0.539–2.669; *P* = 0.655) did not differ between the adjuvant and observation groups. Advanced T stage and positive resection margin were independently associated with inferior DFS and OS on multivariate analysis.

**Conclusions:**

AC did not improve DFS and OS for patients with ypT0-3N0 rectal cancer after preoperative CRT followed by TME in this cohort study. The confirmative role of AC in locally advanced rectal cancer should be evaluated in prospective randomized trials with a larger sample size.

**Electronic supplementary material:**

The online version of this article (10.1186/s12885-017-3624-7) contains supplementary material, which is available to authorized users.

## Background

Total mesorectal excision (TME) has substantially contributed to improvement in loco-regional recurrence rates and survival for patients with rectal cancer [1]. In addition, benefits in local disease control, toxicity, and sphincter preservation have been achieved by preoperative chemoradiation therapy (CRT), which is the currently standard management for locally advanced rectal cancer (LARC) [2]. Therefore, the focus on improving outcomes has changed from lowering the local recurrence rate to reducing distant recurrence, which still occurs in approximately one-third of patients treated surgically with curative intent [3]. In colon cancer, adjuvant single agent 5-fluorouracil (5-FU) chemotherapy has led to an increase in overall survival (OS), of approximately 10% for patients with American Joint Committee on Cancer (AJCC) stage III disease and a further 5% by adding oxaliplatin [4–7]. Based on this background, many researchers have tried to extrapolate the benefits of adjuvant chemotherapy (AC) for colon cancer to the treatment of rectal cancer. Even if a surgical specimen obtained from radical resection reveals a complete response without any viable tumor cells, the patient is expected to complete 4–6 months of AC based on their clinical stage, which was estimated before preoperative treatment [8, 9].

However, the efficacy of AC in patients with LARC after preoperative CRT and TME has not been documented to the same extent, and the clinical benefit remains controversial [10]. In this context, international and national treatment guidelines differ in their recommendations regarding to AC in LARC [11, 12]. The initial results of the EORTC 22921 trial indicated that only patients with a good prognosis (ypT0–2) benefited from AC [13]. However, the final results indicated that adjuvant 5-FU-based chemotherapy after preoperative radiotherapy with or without chemotherapy did not improve disease-free survival (DFS) and OS in all patients, including patients with a good prognosis (ypT0–2) [14]. In contrast, patients with a high risk for recurrence (yp stage III) benefited from adding oxaliplatin to 5-FU as AC after preoperative 5-FU-based CRT and TME [15]. With these heterogeneous results about the role of AC, we aimed to investigate the value of AC with fluoropyrimidine mono-therapy after preoperative CRT and TME in ypT0-3N0 patients, who are considered to have a good prognosis.

## Methods

### Patients and pretreatment evaluation

Patients who were diagnosed with LARC, were treated with preoperative CRT and TME, and had ypT0-3N0M0 as the final pathologic diagnosis at Yonsei Cancer Center between January 2001 and December 2013 were included. Patients who underwent trans-anal excision and received AC with oxaliplatin were excluded.

Pathologic diagnosis by biopsy was performed for all patients before treatment. To determine the clinical stage, the pretreatment evaluation involved a physical examination including a digital rectal examination; carcinoembryonic antigen (CEA); abdomino-pelvic computed tomography (CT); chest CT; rectal magnetic resonance imaging; and positron emission tomography (PET)-CT, when there was a suspicion of distant metastasis. Clinical and pathologic staging were determined according to the AJCC TNM staging system, 7th edition [16].

### Treatment and follow-up

Preoperative radiation therapy involved a total of 45–50.4 Gray radiation delivered in 25–28 fractions to the tumor and drained lymph node. Preoperative chemotherapy with concurrent radiotherapy included 5-FU administered as a 400-mg/m^2^ bolus and leucovorin administered as a 20-mg/m^2^ bolus during the first and last weeks of radiotherapy or 850-mg/m^2^ capecitabine twice a day during the entire period of radiotherapy. Surgical resection with TME was performed 4–8 weeks after completion of the CRT.

AC consisted of 5-FU administered as a 400-mg/m^2^ bolus and leucovorin administered as a 20-mg/m^2^ bolus on days 1–5 every 28 days for 4 cycles or 1250-mg/m^2^ capecitabine twice a day on days 1–14 every 21 days for 5 cycles. The chemotherapeutic agents were the same as those used in the preoperative CRT.

Patients were followed at 3-month intervals during the first 2 years after surgery, at 6-month intervals during the next 3 years, and annually thereafter. At each visit during the regular follow-ups, a serum CEA assay was performed. Abdomino-pelvic CT was performed at 6-month intervals, chest CT was performed at 12-month intervals, and both were performed annually after 5 years. If recurrence was suspected, the follow-up examinations included a clinical evaluation, physical examination, serum CEA assay, chest CT, abdomino-pelvic CT, colonoscopy, and PET, as appropriate. Recurrence was determined using clinical and radiological examinations or histological assessment.

### Statistical analysis

To evaluate the benefit of AC for patients with LARC treated with preoperative CRT and TME, we compared survival between the patients with AC (adjuvant group) and those without AC (observation group). To reduce the effect of treatment-selection bias and simulate the effects of randomization, propensity score matching was used. Propensity scores were estimated using a logistic regression model based on age, sex, tumor location, histologic differentiation, pretreatment CEA level, surgical procedure, pathologic stage, number of retrieved lymph nodes, lymphovascular or perineural invasion, and margin involvement. One-to-one matching without replacement was performed using a 0.2 caliper width, and the resulting score-matched pairs were used in subsequent analyses, as indicated.

The statistical significance of differences was assessed using the Chi-square test for categorical variables and Wilcoxon rank sum test for continuous variables unless specifically mentioned. OS was defined as the time from the date of surgery to the date of death from any cause. DFS was defined as the time from the date of surgery to the detection of recurrent disease or death, whichever occurred first. Survival curves were generated using the Kaplan-Meier method, and survival was compared using Cox regression analysis. To identify the subpopulations that benefited from AC, subgroup analysis was performed by stratifying patients according to patient demographics and tumor characteristics in the entire sample as well as the propensity score-matched cohort. All analyses were conducted with the statistical program R (R Foundation for Statistical Computing, Vienna, Austria). All *P*-values are two-sided, and *P* < 0.05 was used to denote statistical significance.

## Results

### Patient characteristics

**Fig. 1:**
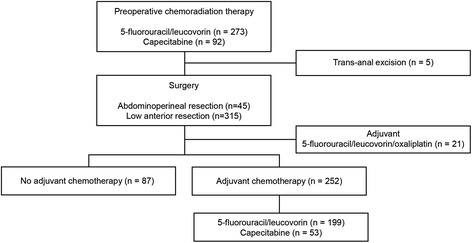
Flowchart of the study population

Of the 365 patients with LARC (radiological T3–4 or N+) who underwent neoadjuvant CRT with 5-FU or capecitabine followed by TME, 5 patients who underwent a trans-anal excision and 21 patients who sequentially received AC with oxaliplatin were excluded (Fig. 1). Therefore, the analyses included 339 patients with ypT0-3N0 primary adenocarcinoma of the rectum.

The baseline characteristics of all patients are presented in Table 1. The median number of harvested lymph nodes was 12 (interquartile range [IQR], 9–17), and total pathologic complete remission (ypT0N0) was achieved in 90 patients (26.5%). Of the 399 patients, 87 patients (25.7%) did not receive AC. Compared with patients who did not receive AC, patients who received AC were younger, had a more advanced pathologic stage, and experienced a poor response to preoperative CRT. Other clinicopathologic characteristics were similar between the adjuvant and observation groups.

**Table 1:** Baseline characteristics of patients with ypT0-3N0 rectal cancer

		Before matching			After matching		
	Overall *n* = 339 (%)	No AC *n* = 87 (%)	AC *n* = 252 (%)	*p-*value	No AC *n* = 87 (%)	AC *n* = 87 (%)	*p-*value
Median age (range), years	59.6 (27.2–85.7)	65.9 (36.0–85.7)	58.3 (27.2–84.6)	<0.001	65.9 (36.0–85.7)	63.7 (39.1–84.6)	0.251
Sex				0.752			0.602
Male	246 (72.6)	62 (71.3)	184 (73.0)		62 (71.3)	59 (67.8)	
Female	93 (27.4)	25 (28.7)	68 (27.0)		25 (28.7)	28 (32.2)	
Distance from AV (cm)							0.342
≥ 10.0	35 (10.3)	8 (9.2)	27 (10.7)		8 (9.2)	9 (10.3)	
5.0–9.9	140 (41.3)	33 (37.9)	107 (42.5)		33 (37.9)	40 (46.0)	
< 5.0	164 (48.4)	46 (52.9)	118 (46.8)		46 (52.9)	38 (43.7)	
Differentiation				0.176			0.300
Well	70 (20.6)	17 (19.5)	53 (21.0)		17 (19.5)	20 (23.0)	
Moderate	248 (73.2)	61 (70.1)	187 (74.2)		61 (70.1)	62 (71.3)	
Poor, mucinous	21 (6.2)	9 (10.3)	12 (4.8)		9 (10.3)	5 (5.7)	
Pretreatment CEA (ng/mL)				0.621			0.413
< 5	214 (63.1)	53 (60.9)	161 (63.9)		53 (60.9)	58 (66.7)	
≥ 5	125 (36.9)	34 (39.1)	91 (36.1)		34 (39.1)	29 (33.3)	
Surgical procedure				0.573			0.350
LAR	298 (87.9)	75 (86.2)	232 (88.5)		75 (86.2)	79 (90.8)	
APR	41 (12.1)	12 (13.8)	29 (11.5)		12 (13.8)	8 (9.2)	
Stage				<0.001			0.383
ypT0	90 (26.5)	40 (46.0)	50 (19.8)		40 (46.0)	37 (42.5)	
ypT1	19 (5.6)	6 (6.9)	13 (5.2)		6 (6.9)	4 (4.6)	
ypT2	96 (28.3)	25 (28.7)	71 (28.2)		25 (28.7)	26 (29.9)	
ypT3	134 (39.5)	16 (18.4)	118 (46.8)		16 (18.4)	20 (23.0)	
LN dissected				0.146			0.538
< 12	145 (42.8)	43 (49.4)	102 (40.5)		43 (49.4)	39 (44.8)	
≥ 12	194 (57.2)	44 (50.6)	150 (59.5)		44 (50.6)	48 (55.2)	
LVI/PNI				0.812			0.657
Negative	278 (82.0)	71 (81.6)	207 (82.1)		71 (81.6)	80 (92.0)	
Positive	15 (4.4)	3 (3.4)	12 (4.8)		3 (3.5)	2 (2.3)	
NA	46 (13.6)	13 (14.9)	33 (13.1)		13 (14.9)	5 (5.8)	
Margin				0.677			1.000
Negative	329 (97.1)	85 (97.7)	244 (96.8)		85 (97.7)	85 (97.7)	
Positive	10 (2.9)	2 (2.3)	8 (3.2)		2 (2.3)	2 (2.3)	
Mandard regression grade				<0.001			0.926
Grade 1	90 (26.5)	40 (46.0)	50 (19.8)		40 (46.0)	37 (42.5)	
Grade 2	90 (26.5)	17 (19.5)	73 (29.0)		17 (19.5)	22 (25.3)	
Grade 3	78 (23.0)	11 (12.6)	67 (26.6)		11 (12.6)	15 (17.2)	
Grade 4	36 (10.6)	6 (6.9)	30 (11.9)		6 (6.9)	8 (9.2)	
NA	45 (13.3)	13 (14.9)	32 (12.7)		13 (14.9)	5 (5.8)	

*AC* adjuvant chemotherapy, *AV* anal verge, *CEA* carcinoembryonic antigen, *LAR* lower anterior resection, *APR* abdomino-perineal resection, *LN* lymph node, *LVI* lymphovascular invasion, *PNI* perineural invasion, *NA* not assessed

### Oncologic outcomes

**Fig. 2:**
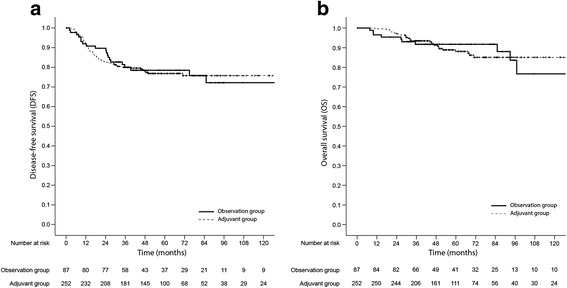
Kaplan-Meier survival plots of disease-free survival (**a**) and overall survival (**b**) based on the receipt of adjuvant chemotherapy in the entire sample

The mean follow-up duration was 70.7 months (95% confidence interval, 65.9–75.5 months), and the duration was similar between the two groups (*P* = 0.650). Local recurrence and systemic recurrence occurred in 23 patients (6.8%) and 57 patients (16.8%), respectively. The lung was the most common site of distant metastasis (37 patients), followed by the liver (16 patients) and distant lymph nodes (7 patients). A total of 40 patients died, and 28 deaths occurred due to cancer progression. No treatment-related mortality was reported. In the multivariate Cox regression analysis, old age (>70 years old), abdomino-perineal resection, advanced pathologic stage (ypT stage), lymphovascular or perineural invasion, and a positive resection margin were associated with inferior DFS. In addition, patients who were older, had an advanced pathologic stage, and had a positive resection margin showed poor OS in the multivariate analysis (Table 2). However, there were no significant differences in DFS and OS based on AC (Fig. 2). The 5-year DFS were 78.0% in the observation group and 76.8% in the adjuvant group (hazard ratio [HR], 0.921; 95% confidence interval [CI], 0.562–1.507; *P* = 0.742). AC did not confer a benefit in terms of both local recurrence (HR, 1.583; 95% CI, 0.538–4.652; *P* = 0.404), and systemic recurrence (HR, 1.070; 95% CI, 0.585–1.956; *P* = 0.825). The 5-year OS were 91.6% in the observation group and 88.1% in the adjuvant group (HR, 0.835; 95% CI, 0.423–1.648; *P* = 0.603).

**Table 2:** Factors associated with disease-free survival and overall survival in the entire sample of patients with ypT0-3N0 rectal cancer

	Disease-free survival				Overall survival			
	Univariate analysis		Multivariate analysis		Univariate analysis		Multivariate analysis	
	HR (95% CI)	*p-*value	HR (95% CI)	*p-*value	HR (95% CI)	*p-*value	HR (95% CI)	*p-*value
Age, years		0.002		0.013		0.001		<0.001
< 70	1		1		1		1	
≥ 70	2.091 (1.299–3.366)		1.944 (1.148–3.293)		3.026 (1.584–5.780)		3.606 (1.848–7.038)	
Sex		0.226				0.051		
Female	1				1			
Male	1.393 (0.814–2.384)				2.549 (0.998–6.511)			
Distance from AV (cm)		0.122				0.302		
≥ 10.0	1				1			
5.0–9.9	0.754 (0.340–1.674)				0.683 (0.220–2.122)			
< 5.0	1.256 (0.591–2.668)				1.184 (0.407–3.443)			
Differentiation		0.646				0.327		
Well	1				1			
Moderate	1.045 (0.591–1.848)				1.103 (0.480–2.536)			
Poor, mucinous	1.493 (0.607–3.677)				2.202 (0.692–7.010)			
Pretreatment CEA (ng/mL)		0.005		0.057		0.088		
< 5	1		1		1			
≥ 5	1.877 (1.207–2.918)		1.628 (0.985–2.692)		1.717 (0.923–3.196)			
Surgical procedure		0.001		<0.001		0.008		0.059
LAR	1		1		1		1	
APR	2.492 (1.480–4.196)		3.919 (1.996–7.697)		2.538 (1.272–5.064)		2.007 (0.974–4.135)	
Stage		0.005		0.047		0.007		0.007
ypT0	1		1		1		1	
ypT1	2.234 (0.688–7.256)		1.876 (0.505–6.972)		5.049 (1.018–25.046)		4.853 (0.977–24.109)	
ypT2	2.317 (1.066–5.038)		2.661 (1.193–5.936)		1.872 (0.483–7.261)		1.488 (0.379–5.842)	
ypT3	3.555 (1.732–7.295)		2.917 (1.354–6.284)		5.340 (1.612–17.684)		4.742 (1.419–15.843)	
LN dissected		0.119				0.100		
≥ 12	1				1			
< 12	1.421 (0.913–2.212)				1.691 (0.904–3.166)			
LVI/PNI		0.005		0.023		0.445		
Negative	1		1		1			
Positive	3.047 (1.391–6.678)		2.606 (1.144–5.938)		1.753 (0.415–7.401)			
Margin		<0.001		<0.001		<0.001		<0.001
Negative	1		1		1		1	
Positive	9.165 (4.653–18.050)		6.348 (2.786–14.467)		10.374 (4.546–23.673)		7.933 (3.351–18.779)	
Mandard regression grade		0.004				0.050		
Grade 1	1				1			
Grade 2	2.503 (1.146–5.466)				3.040 (0.823–11.230)			
Grade 3	3.324 (1.538–7.185)				3.467 (0.938–12.809)			
Grade 4	4.434 (1.894–10.378)				6.702 (1.731–25.946)			
Adjuvant chemotherapy		0.742				0.603		
No	1				1			
Yes	0.921 (0.562–1.507)				0.835 (0.423–1.648)			

*HR* hazard ratio, *CI* confidence interval, *AV* anal verge, *CEA* carcinoembryonic antigen, *LAR* lower anterior resection, *APR* abdomino-perineal resection, *LN* lymph node, *LVI* lymphovascular invasion, *PNI* perineural invasion

### Propensity score-matched analysis

**Fig. 3:**
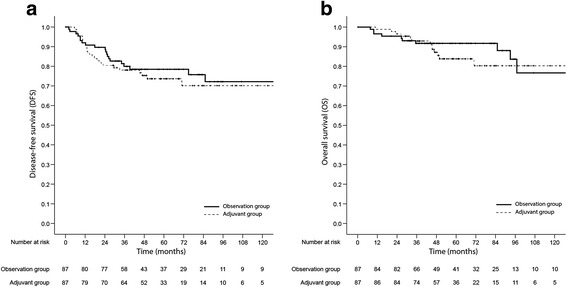
Kaplan-Meier survival plots of disease-free survival (**a**) and overall survival (**b**) based on receipt of adjuvant chemotherapy in the propensity score-matched cohort

We conducted the propensity score-matched analysis because the patients treated with AC were younger, had a more advanced pathologic stage, and experienced a poor response to preoperative CRT and these parameters were independent poor prognostic factors in the Cox regression analysis. The propensity score matching resulted in 87 matched pairs, for a total of 174 patients. The patient characteristics were nearly balanced between the two groups (Table 1). In the propensity score-matched cohort, there were also no significant differences in DFS and OS based on AC (Fig. 3). The 5-year DFS were 78.0% in the observation group and 73.7% in the adjuvant group (HR, 1.129; 95% CI, 0.626–2.035; *P* = 0.688). In addition, both local recurrence (HR, 2.206; 95% CI, 0.679–7.165; *P* = 0.188) and systemic recurrence (HR, 1.089; 95% CI, 0.526–2.258; *P* = 0.818) did not differ between the two groups. The 5-year OS were 91.6% in the observation group and 83.8% in the adjuvant group (HR, 1.200; 95% CI, 0.539–2.669; *P* = 0.655). In the propensity score-matched cohort, advanced pathologic stage and positive resection margin were associated with both inferior DFS and OS (Table 3).

**Table 3:** Factors associated with disease-free survival and overall survival in the cohort of propensity score-matched patients with ypT0-3N0 rectal cancer

	Disease-free survival				Overall survival			
	Univariate analysis		Multivariate analysis		Univariate analysis		Multivariate analysis	
	HR (95% CI)	*p-*value	HR (95% CI)	*p-*value	HR (95% CI)	*p-*value	HR (95% CI)	*p-*value
Age, years		0.038		0.382		0.025		0.269
< 70	1		1		1		1	
≥ 70	1.873 (1.036–3.386)		1.324 (0.706–2.483)		2.513 (1.125–5.609)		1.598 (0.695–3.673)	
Sex		0.220				0.030		0.075
Female	1				1	1	1	
Male	1.553 (0.769–3.138)				4.951 (1.167–21.005)		3.782 (0.875–16.352)	
Distance from AV (cm)		0.511				0.416		
≥ 10.0	1				1			
5.0–9.9	0.669 (0.256–1.745)				0.510 (0.146–1.784)			
< 5.0	0.956 (0.383–2.391)				0.897 (0.281–2.862)			
Differentiation		0.896				0.464		
Well	1				1			
Moderate	0.842 (0.411–1.726)				0.579 (0.233–1.439)			
Poor, mucinous	0.871 (0.271–2.803)				0.868 (0.220–3.432)			
Pretreatment CEA (ng/mL)		0.014		0.039		0.348		
< 5	1		1		1			
≥ 5	2.075 (1.156–3.727)		1.912 (1.033–3.540)		1.461 (0.662–3.226)			
Surgical procedure		0.039		0.339		0.033		0.225
LAR	1		1		1		1	
APR	2.133 (1.038–4.384)		1.465 (0.670–3.206)		2.615 (1.082–6.319)		1.764 (0.705–4.411)	
Stage		0.003		0.019		0.002		0.007
ypT0	1		1		1		1	
ypT1	4.239 (1.299–13.831)		3.461 (1.017–11.778)		10.163 (2.033–50.817)		6.894 (1.343–35.381)	
ypT2	2.388 (1.040–5.485)		1.893 (0.808–4.434)		2.243 (0.555–9.059)		1.647 (0.387–7.005)	
ypT3	4.438 (1.963–10.032)		3.517 (1.528–8.093)		7.533 (2.114–26.848)		5.782 (1.588–21.051)	
LN dissected		0.729				0.946		
≥ 12	1				1			
< 12	0.901 (0.500–1.624)				1.028 (0.467–2.259)			
LVI/PNI		0.294				0.591		
Negative	1				1			
Positive	2.149 (0.516–8.955)				0.047 (0.000–3244.523)			
Margin		<0.001		0.001		<0.001		0.005
Negative	1		1		1		1	
Positive	8.956 (3.131–25.613)		7.193 (2.234–23.156)		10.375 (3.022–35.623)		7.035 (1.806–27.404)	
Mandard regression grade		0.006				0.039		
Grade 1	1				1			
Grade 2	3.086 (1.319–7.222)				5.268 (1.398–19.860)			
Grade 3	4.542 (1.880–10.970)				4.113 (0.920–18.379)			
Grade 4	3.771 (1.263–11.261)				8.159 (1.825–36.482)			
Adjuvant chemotherapy		0.688				0.655		
No	1				1			
Yes	1.129 (0.626–2.035)				1.200 (0.539–2.669)			

*HR* hazard ratio, *CI* confidence interval, *AV* anal verge, *CEA* carcinoembryonic antigen, *LAR* lower anterior resection, *APR* abdomino-perineal resection, *LN* lymph node, *LVI* lymphovascular invasion, *PNI* perineural invasion

### Subgroup analysis of the benefit of adjuvant chemotherapy

In general, AC was not associated with improved DFS and OS in the entire sample (Additional file 1: Table S1) or propensity score-matched patient cohort (Additional file 1: Table S2). However, AC was associated with poor DFS and OS among patients older than 70 years in both study subsets.

## Discussion

In this study, we evaluated the outcomes of LARC for patients with ypT0-3N0, who are considered to have a relatively good prognosis, after preoperative CRT followed by TME, based on receipt of AC. AC did not improve survival in these patients. Because the analysis was conducted using data from a prospectively collected tumor registry, the adjuvant group was younger, had a more advanced pathologic stage, and experienced a poor response to preoperative CRT, compared with the observation group. Even after propensity score matching for these reasons, AC was also not associated with improved outcomes in terms of DFS and OS in the propensity score-matched cohort. We were also not able to identify any specific subpopulations that benefited from AC. These results were comparable with those from previous studies regarding the roles of AC in LARC after preoperative CRT or radiotherapy [17–20].

Before the era of preoperative CRT and TME, AC was associated with improved outcomes in rectal cancer [10, 21]. As loco-regional recurrence rates have recently decreased after the introduction of TME, reduction in distant metastases has become more important in rectal cancer treatment, similar to colon cancer. In addition, substantial improvements have been achieved recently in the management of rectal cancer after the introduction of preoperative CRT and TME. However, the long-term results of the EORTC 22921 [14], CHRONICLE [22], I-CNR-RT [23], PROCTOR/SCRIPT [3], and QUASAR trials [24] are controversial regarding the benefits of AC in patients with LARC after preoperative CRT or radiotherapy followed by surgery. Although the rationale for AC after preoperative CRT was largely extrapolated from the results obtained with colon cancer, the clinical benefit of AC in rectal cancer needs to be validated, considering the different treatment modalities, recurrence patterns, and tumor biology [25, 26]. No conclusive evidence favoring AC in LARC after preoperative CRT and TME currently exists [27].

For colon cancer, the benefit of AC has been clearly demonstrated for patients with stage III disease in multiple clinical trials and meta-analyses [4–7]. However, the benefit of AC in stage II colon cancers is less certain [28, 29]. For patients with stage II T4 colon cancer, AC was associated with improved survival [30]. Therefore, we evaluated whether AC is needed in patients with LARC with ypT0-3N0 after preoperative CRT as well as the subgroup(s) that benefit from AC. However, AC did not appear to benefit any specific subgroup.

There are several possible reasons for a lack of clinical benefit from AC in patients with LARC and ypT0-3N0 after preoperative CRT and TME. First, AC is effective for patients who have a poor prognosis, such as those with stage III or T4 [30]. According to the ADORE trial, which examined the role of oxaliplatin, fluorouracil, and leucovorin as AC for LARC, survival was improved with AC in patients with postoperative pathological stage III disease but not in patients with stage II disease [15]. Second, there are embryological, anatomical, and physiological differences between colon and rectal cancers. Miscosatellite instability and *BRAF* mutation are important prognostic factors but are detected less often in rectal cancer than in colon cancer. A meta-analysis by Breugom et al. suggested that AC might benefit patients with a tumor located 10–15 cm from the anal verge [31], and theoretically, a tumor arising above the peritoneal reflection is more likely to undergo distant spread [32]. Last, the lack of benefit with AC might be attributed to poor compliance. Only 42.9% of participants in the EORTC 22921 trial [13], 43% of participants in the CHRONICLE trial [22], and 55% of the participants who received 3–6 courses of AC in the I-CNR-RT trial [23] benefited from AC.

The researchers of the QUASAR trial identified the patient subgroups that were more likely to benefit from AC: <70 years old, receipt of chemotherapy every 4 weeks, and <6 weeks from surgery to AC [24]. However, we did not identify a subgroup that benefited from AC in the current study, although old age was associated with worse prognosis with AC. Therefore, routine use of AC should be evaluated carefully, considering not only the patient characteristics, such as age and comorbidities, but also tumor characteristics, such as distance from the anal verge and the optimal chemotherapy regimen and duration.

This study has certain limitations. First, the analysis was based on data that were prospectively collected in a tumor registry. Therefore, the baseline characteristics differed, although we corrected this using propensity score-matched analysis. In addition, immortal time bias, caused by a period of time during which events cannot occur [33], could act as a confounding factor. However, we found no definite differences in early recurrence or death as well as DFS and OS at different cut-off times after surgery between the adjuvant and observation groups in this study (Additional file 1: Tables S3 and S4). Also, this bias has clearly not affected the conclusion, because immortal time bias would tend to favor the adjuvant chemotherapy arm if present. Second, this study was conducted with a relatively small sample size, thus underpowered to ascertain the effect of adjuvant chemotherapy. However, the effect size found in the cohort of propensity-matched patients was 1.129 (HF for DFS), which is the opposite direction of the benefit from adjuvant chemotherapy as well as seems to be clinically irrelevant. Furthermore, statistical power for detecting such HR is less than 10%, which means that a huge sample size is needed to show statistical significance. Third, the results were derived from a single tertiary center, potentially lacking the external validation.

## Conclusions

In summary, LARC patients with ypT0-3N0 did not benefit from AC after preoperative CRT and TME, which supports the findings of previous studies investigating the role of AC after preoperative CRT and TME and the conclusions of meta-analyses. However, there are conflicting results about the use of AC from many studies with diverse patient populations. Based on this context, a more intensive investigation is needed to evaluate the potential advantages and drawbacks of AC in the era of preoperative CRT and TME. Moreover, future studies should focus on identifying patient subpopulations that benefit from AC.

## Additional file

Additional file 1: Table S1. Effect of adjuvant chemotherapy on disease-free survival and overall survival by patient demographics and tumor characteristics in the entire sample of patients. **Table S2.** Effect of adjuvant chemotherapy on disease-free survival and overall survival by patient demographics and tumor characteristics in the cohort of propensity score-matched patients. **Table S3.** Recurrence or death events at different times after surgery. Comparisons were done by Fisher’s exact test. **Table S4.** Effect of adjuvant chemotherapy on disease-free survival and overall survival by restricting analysis to patients who remained event-free at different times after surgery. (DOCX 30 kb)

## Abbreviations

5-FU: 5-fluorouracil
AC: Adjuvant chemotherapy
AJCC: American Joint Committee on Cancer
CEA: Carcinoembryonic antigen
CI: Confidence interval
CRT: Chemoradiation therapy
CT: Computed tomography
DFS: Disease-free survival
HR: Hazard ratio
IQR: Interquartile range
LARC: Locally advanced rectal cancer
OS: Overall survival
PET: Positron emission tomography
TME: Total mesorectal excision

## References

[1] Heald RJ, Ryall RD (1986). Recurrence and survival after total mesorectal excision for rectal cancer. Lancet.

[2] Sauer R, Becker H, Hohenberger W, Rodel C, Wittekind C, Fietkau R (2004). Preoperative versus postoperative chemoradiotherapy for rectal cancer. N Engl J Med.

[3] Breugom AJ, van Gijn W, Muller EW, Berglund A, van den Broek CB, Fokstuen T (2015). Adjuvant chemotherapy for rectal cancer patients treated with preoperative (chemo)radiotherapy and total mesorectal excision: a Dutch Colorectal Cancer Group (DCCG) randomized phase III trial. Ann Oncol.

[4] Andre T, Boni C, Navarro M, Tabernero J, Hickish T, Topham C (2009). Improved overall survival with oxaliplatin, fluorouracil, and leucovorin as adjuvant treatment in stage II or III colon cancer in the MOSAIC trial. J Clin Oncol.

[5] Kuebler JP, Wieand HS, O'Connell MJ, Smith RE, Colangelo LH, Yothers G (2007). Oxaliplatin combined with weekly bolus fluorouracil and leucovorin as surgical adjuvant chemotherapy for stage II and III colon cancer: results from NSABP C-07. J Clin Oncol.

[6] Shah MA, Renfro LA, Allegra CJ, Andre T, de Gramont A, Schmoll HJ (2016). Impact of patient factors on recurrence risk and time dependency of oxaliplatin benefit in patients with colon cancer: analysis from modern-era adjuvant studies in the adjuvant colon cancer end points (ACCENT) database. J Clin Oncol.

[7] Sargent D, Sobrero A, Grothey A, O'Connell MJ, Buyse M, Andre T (2009). Evidence for cure by adjuvant therapy in colon cancer: observations based on individual patient data from 20,898 patients on 18 randomized trials. J Clin Oncol.

[8] Kim NK, Hur H (2015). New perspectives on predictive biomarkers of tumor response and their clinical application in preoperative chemoradiation therapy for rectal cancer. Yonsei Med J.

[9] Huh JW, Lee WY, Kim SH, Park YA, Cho YB, Yun SH (2015). Immunohistochemical detection of p53 expression in patients with preoperative chemoradiation for rectal cancer: association with prognosis. Yonsei Med J.

[10] Petersen SH, Harling H, Kirkeby LT, Wille-Jorgensen P, Mocellin S. Postoperative adjuvant chemotherapy in rectal cancer operated for cure. Cochrane Database Syst Rev. 2012. doi:10.1002/14651858.CD004078.pub2:Cd004078. 10.1002/14651858.CD004078.pub2 PMC6599875 22419291

[11] Glimelius B, Tiret E, Cervantes A, Arnold D (2013). Rectal cancer: ESMO clinical practice guidelines for diagnosis, treatment and follow-up. Ann Oncol.

[12] van de Velde CJ, Boelens PG, Borras JM, Coebergh JW, Cervantes A, Blomqvist L (2014). EURECCA colorectal: multidisciplinary management: European consensus conference colon & rectum. Eur J Cancer.

[13] Collette L, Bosset JF, den Dulk M, Nguyen F, Mineur L, Maingon P (2007). Patients with curative resection of cT3-4 rectal cancer after preoperative radiotherapy or radiochemotherapy: does anybody benefit from adjuvant fluorouracil-based chemotherapy? A trial of the European Organisation for Research and Treatment of Cancer Radiation Oncology Group. J Clin Oncol.

[14] Bosset JF, Calais G, Mineur L, Maingon P, Stojanovic-Rundic S, Bensadoun RJ (2014). Fluorouracil-based adjuvant chemotherapy after preoperative chemoradiotherapy in rectal cancer: long-term results of the EORTC 22921 randomised study. Lancet Oncol.

[15] Hong YS, Nam BH, Kim KP, Kim JE, Park SJ, Park YS (2014). Oxaliplatin, fluorouracil, and leucovorin versus fluorouracil and leucovorin as adjuvant chemotherapy for locally advanced rectal cancer after preoperative chemoradiotherapy (ADORE): an open-label, multicentre, phase 2, randomised controlled trial. Lancet Oncol.

[16] Edge SB, Compton CC (2010). The American Joint Committee on Cancer: the 7th edition of the AJCC cancer staging manual and the future of TNM. Ann Surg Oncol.

[17] Kiran RP, Kirat HT, Burgess AN, Nisar PJ, Kalady MF, Lavery IC (2012). Is adjuvant chemotherapy really needed after curative surgery for rectal cancer patients who are node-negative after neoadjuvant chemoradiotherapy?. Ann Surg Oncol.

[18] Huh JW, Kim HR (2009). Postoperative chemotherapy after neoadjuvant chemoradiation and surgery for rectal cancer: is it essential for patients with ypT0-2N0?. J Surg Oncol.

[19] Jung KU, Kim HC, Park JO, Park YS, Park HC, Choi DH (2015). Adjuvant chemotherapy after neoadjuvant chemoradiation and curative resection for rectal cancer: is it necessary for all patients?. J Surg Oncol.

[20] Park IJ, Kim DY, Kim HC, Kim NK, Kim HR, Kang SB (2015). Role of adjuvant chemotherapy in ypT0-2N0 patients treated with preoperative chemoradiation therapy and radical resection for rectal cancer. Int J Radiat Oncol Biol Phys.

[21] Fisher B, Wolmark N, Rockette H, Redmond C, Deutsch M, Wickerham DL (1988). Postoperative adjuvant chemotherapy or radiation therapy for rectal cancer: results from NSABP protocol R-01. J Natl Cancer Inst.

[22] Glynne-Jones R, Counsell N, Quirke P, Mortensen N, Maraveyas A, Meadows HM (2014). Chronicle: results of a randomised phase III trial in locally advanced rectal cancer after neoadjuvant chemoradiation randomising postoperative adjuvant capecitabine plus oxaliplatin (XELOX) versus control. Ann Oncol.

[23] Sainato A, Cernusco Luna Nunzia V, Valentini V, De Paoli A, Maurizi ER, Lupattelli M (2014). No benefit of adjuvant Fluorouracil Leucovorin chemotherapy after neoadjuvant chemoradiotherapy in locally advanced cancer of the rectum (LARC): Long term results of a randomized trial (I-CNR-RT). Radiother Oncol.

[24] Gray R, Barnwell J, McConkey C, Hills RK, Williams NS, Kerr DJ (2007). Adjuvant chemotherapy versus observation in patients with colorectal cancer: a randomised study. Lancet.

[25] Kapiteijn E, Liefers GJ, Los LC, Kranenbarg EK, Hermans J, Tollenaar RA (2001). Mechanisms of oncogenesis in colon versus rectal cancer. J Pathol.

[26] Birkenkamp-Demtroder K, Olesen SH, Sorensen FB, Laurberg S, Laiho P, Aaltonen LA (2005). Differential gene expression in colon cancer of the caecum versus the sigmoid and rectosigmoid. Gut.

[27] Bujko K, Glynne-Jones R, Bujko M (2010). Does adjuvant fluoropyrimidine-based chemotherapy provide a benefit for patients with resected rectal cancer who have already received neoadjuvant radiochemotherapy? A systematic review of randomised trials. Ann Oncol.

[28] Moertel CG, Fleming TR, Macdonald JS, Haller DG, Laurie JA, Tangen CM (1995). Intergroup study of fluorouracil plus levamisole as adjuvant therapy for stage II/Dukes’ B2 colon cancer. J Clin Oncol.

[29] Schippinger W, Samonigg H, Schaberl-Moser R, Greil R, Thodtmann R, Tschmelitsch J (2007). A prospective randomised phase III trial of adjuvant chemotherapy with 5-fluorouracil and leucovorin in patients with stage II colon cancer. Br J Cancer.

[30] Kumar A, Kennecke HF, Renouf DJ, Lim HJ, Gill S, Woods R (2015). Adjuvant chemotherapy use and outcomes of patients with high-risk versus low-risk stage II colon cancer. Cancer.

[31] Breugom AJ, Swets M, Bosset JF, Collette L, Sainato A, Cionini L (2015). Adjuvant chemotherapy after preoperative (chemo)radiotherapy and surgery for patients with rectal cancer: a systematic review and meta-analysis of individual patient data. Lancet Oncol.

[32] Benzoni E, Terrosu G, Bresadola V, Cerato F, Cojutti A, Milan E (2006). Analysis of clinical outcomes and prognostic factors of neoadjuvant chemoradiotherapy combined with surgery: intraperitoneal versus extraperitoneal rectal cancer. Eur J Cancer Care (Engl).

[33] Suissa S (2008). Immortal time bias in pharmaco-epidemiology. Am J Epidemiol.

