# Supplementary material for: Role of adjuvant chemotherapy in locally advanced rectal cancer with ypT0-3N0 after preoperative chemoradiation therapy and surgery
Source: BMC Cancer. 2017 Sep 2;17:615. doi: 10.1186/s12885-017-3624-7 (PMC5581409; doi:10.1186/s12885-017-3624-7)
Supplement: Additional file 1: Table S1. — Effect of adjuvant chemotherapy on disease-free survival and overall survival by patient demographics and tumor characteristics in the entire sample of patients. Table S2. Effect of adjuvant chemotherapy on disease-free survival and overall survival by patient demographics and tumor characteristics in the cohort of propensity score-matched patients. Table S3. Recurrence or death events at different times after surgery. Comparisons were done by Fisher’s exact test. Table S4. Effect of adjuvant chemotherapy on disease-free survival and overall survival by restricting analysis to patients who remained event-free at different times after surgery. (DOCX 30 kb) [file 12885_2017_3624_MOESM1_ESM.docx]

**Additional file 1**

**Table S1** Effect of adjuvant chemotherapy on disease-free survival and overall survival by patient demographics and tumor characteristics in the entire sample of patients with ypT0-3N0 rectal cancer

|  |  |  |  | Disease-free survival | |  | Overall survival | |
| --- | --- | --- | --- | --- | --- | --- | --- | --- |
|  |  | n = 339 (%) |  | HR (95% CI) | *p-* value |  | HR (95% CI) | *p-* value |
| Age |  |  |  |  |  |  |  |  |
| <70 years |  | 272 (80.2) |  | 0.705 (0.377-1.320) | 0.275 |  | 0.275 (0.253-1.478) | 0.275 |
| ≥70 years |  | 67 (19.8) |  | 3.191 (1.361-7.477) | 0.008 |  | 3.626 (1.131-11.622) | 0.030 |
| Sex |  |  |  |  |  |  |  |  |
| Male |  | 246 (72.6) |  | 1.044 (0.590-1.847) | 0.884 |  | 0.909 (0.435-1.902) | 0.800 |
| Female |  | 93 (27.4) |  | 0.613 (0.226-1.660) | 0.336 |  | 0.484 (0.081-2.909) | 0.428 |
| Distance from AV |  |  |  |  |  |  |  |  |
| ≥10.0 cm |  | 35 (10.3) |  | 0.466 (0.103-2.104) | 0.321 |  | 0.006 (0.000-51.028) | 0.270 |
| 5.0-9.9 cm |  | 140 (41.3) |  | 0.974 (0.389-2.443) | 0.956 |  | 3.128 (0.403-24.267) | 0.275 |
| < 5.0 cm |  | 164 (48.4) |  | 1.112 (0.575-2.147) | 0.753 |  | 0.915 (0.379-2.212) | 0.844 |
| Differentiation |  |  |  |  |  |  |  |  |
| Well |  | 70 (20.6) |  | 1.259 (0.355-4.469) | 0.721 |  | 1.955 (0.235-16.292) | 0.535 |
| Moderate |  | 248 (73.2) |  | 0.865 (0.484-1.544) | 0.624 |  | 0.719 (0.323-1.600) | 0.419 |
| Poor, mucinous |  | 21 (6.2) |  | 1.082 (0.237-4.944) | 0.919 |  | 1.010 (0.165-6.187) | 0.991 |
| Pretreatment CEA |  |  |  |  |  |  |  |  |
| <5 ng/mL |  | 214 (63.1) |  | 1.076 (0.523-2.215) | 0.841 |  | 0.730 (0.290-1.835) | 0.503 |
| ≥5 ng/mL |  | 125 (36.9) |  | 0.849 (0.430-1.676) | 0.637 |  | 1.089 (0.391-3.032) | 0.870 |
| Surgical procedure |  |  |  |  |  |  |  |  |
| LAR |  | 298 (87.9) |  | 0.861 (0.491-1.512) | 0.603 |  | 0.836 (0.367-1.904) | 0.670 |
| APR |  | 41 (12.1) |  | 1.348 (0.482-3.775) | 0.569 |  | 1.041 (0.306-3.537) | 0.949 |
| Stage |  |  |  |  |  |  |  |  |
| ypT0 |  | 90 (26.5) |  | 0.604 (0.162-2.258) | 0.454 |  | 1.325 (0.118-14.838) | 0.819 |
| ypT1 |  | 19 (5.6) |  | 0.569 (0.051-6.330) | 0.696 |  | 0.211 (0.017-2.597) | 0.224 |
| ypT2 |  | 96 (28.3) |  | 0.859 (0.344-2.146) | 0.745 |  | 0.372 (0.079-1.754) | 0.211 |
| ypT3 |  | 134 (39.5) |  | 0.720 (0.320-1.620) | 0.427 |  | 0.608 (0.228-1.619) | 0.319 |
| LN dissected |  |  |  |  |  |  |  |  |
| <12 |  | 145 (42.8) |  | 1.776 (0.818-3.855) | 0.147 |  | 1.823 (0.616-5.393) | 0.278 |
| ≥12 |  | 194 (57.2) |  | 0.542 (0.280-1.046) | 0.068 |  | 0.404 (0.157-1.038) | 0.060 |
| LVI/PNI |  |  |  |  |  |  |  |  |
| Negative |  | 278 (82.0) |  | 1.021 (0.569-1.832) | 0.945 |  | 0.895 (0.378-2.119) | 0.801 |
| Positive |  | 15 (4.4) |  | 1.328 (0.159-11.077) | 0.793 |  | 30.080 (0.000→) | 0.639 |
| Margin |  |  |  |  |  |  |  |  |
| Negative |  | 329 (97.1) |  | 0.875 (0.520-1.475) | 0.617 |  | 0.712 (0.343-1.476) | 0.361 |
| Positive |  | 10 (2.9) |  | 1.293 (0.259-6.444) | 0.754 |  | 2.558 (0.298-21.948) | 0.392 |
| Mandard regression grade |  |  |  |  |  |  |  |  |
| Grade 1 |  | 90 (26.5) |  | 0.604 (0.162-2.258) | 0.454 |  | 1.325 (0.118-14.838) | 0.819 |
| Grade 2 |  | 90 (26.5) |  | 0.749 (0.274-2.046) | 0.573 |  | 0.455 (0.114-1.825) | 0.267 |
| Grade 3 |  | 78 (23.0) |  | 0.673 (0.229-1.979) | 0.471 |  | 0.491 (0.102-2.370) | 0.376 |
| Grade 4 |  | 36 (10.6) |  | 1.117 (0.247-5.048) | 0.885 |  | 1.196 (0.144-9.937) | 0.869 |

*HR* hazard ratio, *CI* confidence interval, *AV* anal verge, *CEA* carcinoembryonic antigen, *LAR* lower anterior resection, *APR* abdomino-perineal resection, *LN* lymph node, *LVI* lymphovascular invasion, *PNI* perineural invasion

**Table S2** Effect of adjuvant chemotherapy on disease-free survival and overall survival by patient demographics and tumor characteristics in the cohort of propensity score-matched patients with ypT0-3N0 rectal cancer

|  |  |  |  | | Disease-free survival | | |  | Overall survival | |
| --- | --- | --- | --- | --- | --- | --- | --- | --- | --- | --- |
|  |  | n = 174 (%) | |  | | HR (95% CI) | *p-* value |  | HR (95% CI) | *p-* value |
| Age |  |  | |  | |  |  |  |  |  |
| <70 years |  | 117 (67.2) | |  | | 0.704 (0.318-1.557) | 0.386 |  | 0.698 (0.229-2.127) | 0.527 |
| ≥70 years |  | 57 (32.8) | |  | | 3.262 (1.304-8.156) | 0.011 |  | 3.754 (1.083-13.014) | 0.037 |
| Sex |  |  | |  | |  |  |  |  |  |
| Male |  | 121 (69.5) | |  | | 1.507 (0.765-2.972) | 0.236 |  | 1.689 (0.720-3.961) | 0.228 |
| Female |  | 53 (30.5) | |  | | 0.525 (0.148-1.870) | 0.321 |  | 0.013 (0.000→) | 0.458 |
| Distance from AV |  |  | |  | |  |  |  |  |  |
| ≥10.0 cm |  | 17 (9.8) | |  | | 0.592 (0.104-3.361) | 0.554 |  | 0.017 (0.000-77.555) | 0.344 |
| 5.0-9.9 cm |  | 73 (41.9) | |  | | 1.323 (0.468-3.741) | 0.598 |  | 4.438 (0.533-36.979) | 0.168 |
| < 5.0 cm |  | 84 (48.3) | |  | | 1.313 (0.579-2.978) | 0.514 |  | 1.290 (0.431-3.860) | 0.649 |
| Differentiation |  |  | |  | |  |  |  |  |  |
| Well |  | 37 (21.3) | |  | | 2.095 (0.541-8.114) | 0.284 |  | 5.434 (0.650-45.398) | 0.118 |
| Moderate |  | 123 (70.7) | |  | | 0.989 (0.484-2.018) | 0.975 |  | 0.743 (0.258-2.138) | 0.581 |
| Poor, mucinous |  | 14 (8.0) | |  | | 0.435 (0.041-4.619) | 0.490 |  | 0.463 (0.040-5.434) | 0.540 |
| Pretreatment CEA |  |  | |  | |  |  |  |  |  |
| <5 ng/mL |  | 111 (63.8) | |  | | 1.445 (0.587-3.555) | 0.423 |  | 1.345 (0.420-4.310) | 0.618 |
| ≥5 ng/mL |  | 63 (36.2) | |  | | 1.153 (0.457-2.907) | 0.758 |  | 1.386 (0.423-4.548) | 0.590 |
| Surgical procedure |  |  | |  | |  |  |  |  |  |
| LAR |  | 154 (88.5) | |  | | 1.074 (0.550-2.097) | 0.835 |  | 1.135 (0.429-3.002) | 0.799 |
| APR |  | 20 (11.5) | |  | | 1.831 (0.523-6.406) | 0.344 |  | 2.131 (0.519-8.747) | 0.294 |
| Stage |  |  | |  | |  |  |  |  |  |
| ypT0 |  | 77 (44.3) | |  | | 0.824 (0.220-3.081) | 0.774 |  | 1.780 (0.159-19.950) | 0.640 |
| ypT1 |  | 10 (5.8) | |  | | 0.274 (0.026-2.878) | 0.280 |  | 1.000 (0.059-16.928) | 1.000 |
| ypT2 |  | 51 (29.3) | |  | | 1.287 (0.459-3.608) | 0.632 |  | 0.662 (0.116-3.765) | 0.641 |
| ypT3 |  | 36 (20.7) | |  | | 1.330 (0.502-3.526) | 0.566 |  | 1.471 (0.471-4.600) | 0.507 |
| LN dissected |  |  | |  | |  |  |  |  |  |
| <12 |  | 82 (47.1) | |  | | 1.718 (0.701-4.209) | 0.236 |  | 1.961 (0.588-6.541) | 0.273 |
| ≥12 |  | 92 (52.9) | |  | | 0.844 (0.373-1.908) | 0.683 |  | 0.742 (0.228-2.418) | 0.621 |
| LVI/PNI |  |  | |  | |  |  |  |  |  |
| Negative |  | 151 (86.8) | |  | | 1.254 (0.646-2.435) | 0.504 |  | 1.371 (0.539-3.489) | 0.508 |
| Positive |  | 5 (2.9) | |  | | 1.225 (0.076-19.862) | 0.887 |  | NA | NA |
| Margin |  |  | |  | |  |  |  |  |  |
| Negative |  | 170 (97.7) | |  | | 1.134 (0.611-2.105) | 0.689 |  | 1.079 (0.460-2.531) | 0.861 |
| Positive |  | 4 (2.3) | |  | | 2.562 (0.225-29.121) | 0.448 |  | 104.944 (0.001→) | 0.439 |
| Mandard regression grade |  |  | |  | |  |  |  |  |  |
| Grade 1 |  | 77 (44.3) | |  | | 0.824 (0.220-3.081) | 0.774 |  | 1.780 (0.159-19.950) | 0.640 |
| Grade 2 |  | 39 (22.4) | |  | | 1.301 (0.425-3.979) | 0.645 |  | 1.248 (0.297-5.241) | 0.762 |
| Grade 3 |  | 26 (14.9) | |  | | 1.267 (0.370-4.332) | 0.706 |  | 0.691 (0.097-4.918) | 0.691 |
| Grade 4 |  | 14 (8.1) | |  | | 1.165 (0.194-7.008) | 0.868 |  | 2.070 (0.214-20.012) | 0.530 |

*HR* hazard ratio, *CI* confidence interval, *AV* anal verge, *CEA* carcinoembryonic antigen, *LAR* lower anterior resection, *APR* abdomino-perineal resection, *LN* lymph node, *LVI* lymphovascular invasion, *PNI* perineural invasion

**Table S3** Recurrence or death events at different times after surgery (comparisons were done by Fisher’s exact test)

|  |  | Before matching | | |  | After matching | | | |
| --- | --- | --- | --- | --- | --- | --- | --- | --- | --- |
|  | Overall n = 339 (%) | No AC n = 87 (%) | AC n = 252 (%) | *p-*value |  | | No AC n = 87 (%) | AC n = 87 (%) | *p-*value |
| Recurrence |  |  |  |  |  | |  |  |  |
| 8 weeks | 0 (0.0) | 0 (0.0) | 0 (0.0) | - |  | | 0 (0.0) | 0 (0.0) | - |
| 6 months | 7 (2.1) | 2 (2.3) | 5 (2.0) | 1.000 |  | | 2 (2.3) | 1 (1.1) | 1.000 |
| 12 months | 25 (7.4) | 6 (6.9) | 19 (7.5) | 1.000 |  | | 6 (6.9) | 8 (9.2) | 0.782 |
| Death |  |  |  |  |  | |  |  |  |
| 8 weeks | 0 (0.0) | 0 (0.0) | 0 (0.0) | - |  | | 0 (0.0) | 0 (0.0) | - |
| 6 months | 0 (0.0) | 0 (0.0) | 0 (0.0) | - |  | | 0 (0.0) | 0 (0.0) | - |
| 12 months | 4 (1.2) | 3 (3.4) | 1 (0.4) | 0.054 |  | | 3 (3.4) | 1 (1.1) | 0.621 |

*AC* adjuvant chemotherapy

**Table S4** Effect of adjuvant chemotherapy on disease-free survival and overall survival by restricting analysis to patients who remained event-free at different times after surgery

|  | Before matching | | |  | After matching | | |
| --- | --- | --- | --- | --- | --- | --- | --- |
|  | HR (95% CI) | | *p-*value |  | HR (95% CI) | | *p-*value |
| Disease-free survival |  |  |  |  |  |  |  |
| 8 weeks | 0.921 (0.562-1.507) | | 0.742 |  | 1.129 (0.626-2.035) | | 0.688 |
| 6 months | 0.927 (0.553-1.555) | | 0.774 |  | 1.196 (0.649-2.204) | | 0.566 |
| 12 months | 0.917 (0.504-1.671) | | 0.778 |  | 1.128 (0.547-2.328) | | 0.744 |
| Overall survival |  |  |  |  |  |  |  |
| 8 weeks | 0.835 (0.423-1.648) | | 0.603 |  | 1.200 (0.539-2.669) | | 0.655 |
| 6 months | 0.818 (0.401-1.672) | | 0.582 |  | 1.217 (0.528-2.806) | | 0.645 |
| 12 months | 1.006 (0.420-2.411) | | 0.989 |  | 1.375 (0.483-3.911) | | 0.551 |

*HR* hazard ratio, *CI* confidence interval
